# RNAspider: a webserver to analyze entanglements in RNA 3D structures

**DOI:** 10.1093/nar/gkac218

**Published:** 2022-03-29

**Authors:** Kamil Luwanski, Vladyslav Hlushchenko, Mariusz Popenda, Tomasz Zok, Joanna Sarzynska, Daniil Martsich, Marta Szachniuk, Maciej Antczak

**Affiliations:** Institute of Computing Science and European Centre for Bioinformatics and Genomics, Poznan University of Technology, Piotrowo 2, 60-965 Poznan, Poland; Institute of Computing Science and European Centre for Bioinformatics and Genomics, Poznan University of Technology, Piotrowo 2, 60-965 Poznan, Poland; Institute of Bioorganic Chemistry, Polish Academy of Sciences, Noskowskiego 12/14, 61-704 Poznan, Poland; Institute of Computing Science and European Centre for Bioinformatics and Genomics, Poznan University of Technology, Piotrowo 2, 60-965 Poznan, Poland; Institute of Bioorganic Chemistry, Polish Academy of Sciences, Noskowskiego 12/14, 61-704 Poznan, Poland; Institute of Computing Science and European Centre for Bioinformatics and Genomics, Poznan University of Technology, Piotrowo 2, 60-965 Poznan, Poland; Institute of Computing Science and European Centre for Bioinformatics and Genomics, Poznan University of Technology, Piotrowo 2, 60-965 Poznan, Poland; Institute of Bioorganic Chemistry, Polish Academy of Sciences, Noskowskiego 12/14, 61-704 Poznan, Poland; Institute of Computing Science and European Centre for Bioinformatics and Genomics, Poznan University of Technology, Piotrowo 2, 60-965 Poznan, Poland; Institute of Bioorganic Chemistry, Polish Academy of Sciences, Noskowskiego 12/14, 61-704 Poznan, Poland

## Abstract

Advances in experimental and computational techniques enable the exploration of large and complex RNA 3D structures. These, in turn, reveal previously unstudied properties and motifs not characteristic for small molecules with simple architectures. Examples include entanglements of structural elements in RNA molecules and knot-like folds discovered, among others, in the genomes of RNA viruses. Recently, we presented the first classification of entanglements, determined by their topology and the type of entangled structural elements. Here, we introduce RNAspider – a web server to automatically identify, classify, and visualize primary and higher-order entanglements in RNA tertiary structures. The program applies to evaluate RNA 3D models obtained experimentally or by computational prediction. It supports the analysis of uncommon topologies in the pseudoknotted RNA structures. RNAspider is implemented as a publicly available tool with a user-friendly interface and can be freely accessed at https://rnaspider.cs.put.poznan.pl/.

## Graphical Abstract

**Graphical Abstract:**
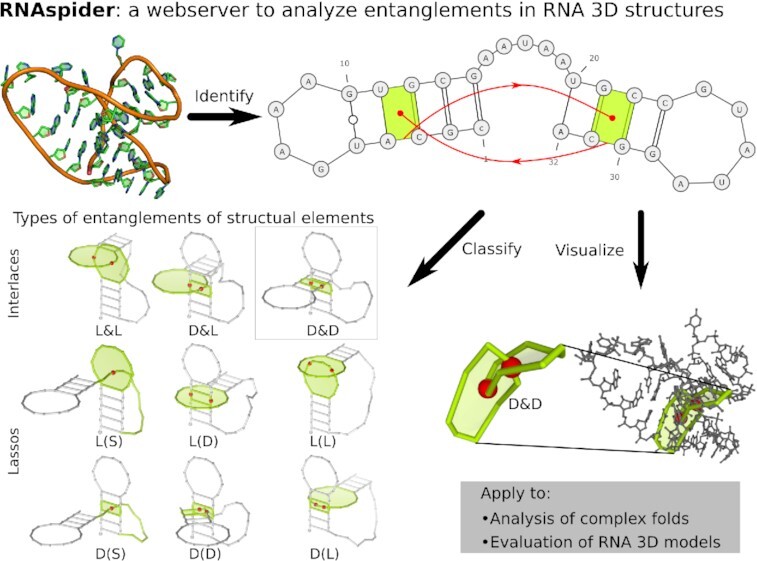
RNAspider: a webserver to analyze entanglements in RNA 3D structures.

## INTRODUCTION

Knowing the structure of RNA is key to understanding many biological processes like gene regulation and translation (1,2). It is also essential in the structure-based drug design and optimizing new therapeutic agents targeting RNA (3,4). Over the years, various structured RNAs have been learned along with their functions. Examples include tRNA (5), riboswitches (6), ribozymes (7) or RNAs in large molecular machines like ribosomes (8) or spliceosomes (9). Also, viruses contain functional RNAs that control critical steps of the replication cycle (10,11). Since the properties and functions of molecules largely depend on structural motifs, tools to study these motifs in known RNA structures and model them correctly by computational methods are much needed.

Determination and prediction of RNA 3D structure is still a challenge. However, better experimental techniques and new computational methods are helping us to learn and analyze it with increasing accuracy, also for large molecules with composite structures. It is becoming possible to capture the specificity of complex structural motifs involving, i.a., large chain fragments or long-range tertiary contacts. Examples include knots and knot-like folds. Knots have been detected in protein structures (12,13) and debated for RNAs (14–16). Their various types – knots, slipknots, links, or lassos – occur when a polymer chain (e.g. protein backbone) threads through itself. Exploring them is aided by protein-centered computational tools (17,18), which can be applied to RNA, although not incorporating base-pairing critical in RNA folding. Knot-like folds have been observed in viral exoribonuclease-resistant RNAs (19,20) and SARS-CoV-2 ribosomal frameshifting element (FSE) (11,21). In these structures, the 5′-end of RNA strand threads through a ring that originates from pseudoknot formation. RNA knot-like motifs display unique mechanical properties and biological functions (22–24).

In our recent paper (25), we have presented the problem of structural element entanglements in RNA 3D structures. We included RNA base pairings in the definition of entanglement, which distinguishes the latter from the topological knot. Based on the secondary structure, we described open and closed structure elements and developed a classification of entanglements considering their topology and the type of tangled elements. We have found various entanglements in RNA 3D models from the RNA-Puzzles benchmark set (26). 38% of over a thousand predictions contained at least one pair of entangled structural elements. We have also identified entanglements within two example PDB structures. Some of these entanglements (e.g. interlaces) appear to be errors in algorithms for the *in silico* modeling of RNA folds or programs to reconstruct the 3D structures from experimental data. Others may be functional motifs. Among the latter is the knot-like fold that – according to our classification – is assigned to higher-order entanglements.

Here, we introduce RNAspider – a webserver to analyze entanglements of structure elements in RNA 3D structures. It identifies primary entanglements and the higher-order ones that involve pseudoknotted base pairs of various orders. It provides the classification of entanglements according to (25), visualizes them in the context of the entire RNA structure on the secondary and tertiary structure view, depicts intersection points of structural elements. Classification of entanglements supports distinguishing between potentially correct and abnormal conformations in the structures studied. Therefore, users can apply RNAspider to preliminary evaluate RNA 3D models generated by various prediction tools and eliminate non-physical ones. The RNAspider algorithm might also supplement the collection of validation and evaluation methods used in the RNA-Puzzles challenges (26–30). Finally, we propose its application in analyzing complex pseudoknotted RNAs – for example, those with unusual, knot-like folds.

## METHOD OUTLINE

RNAspider begins its processing workflow with the validation and preparation of input data. It filters out any non-RNA chain, deletes surplus atoms in modified residues, and removes incomplete nucleotides. Then, it applies the incorporated RNApdbee algorithm (31) to extract the secondary structure and assign orders to pseudoknot-forming base pairs (32). Results of these steps allow the computational engine of RNAspider to partition the input 3D model into open and closed structural elements. The former subset consists of coherent single-stranded fragments not closed by paired nucleotides, e.g. dangling ends. The latter contains elements closed by canonical base pairs – loops and dinucleotide steps.

The system computes a polygonal chain for each open and closed structural element. The chain connects points corresponding to selected atoms and pseudoatoms, i.e. centers of mass of nucleobases involved in base pairing. By definition, a polygonal chain encircles each closed element, which gets covered with a polygon mesh via a recursive triangulation procedure. RNAspider uses Möller–Trumbore algorithm (33) to check every triangle for possible intersection site with a segment of some other polygonal chain. This step allows distinguishing between a puncturing and a punctured element and eventually classifying the entanglement.

**Figure 1.**
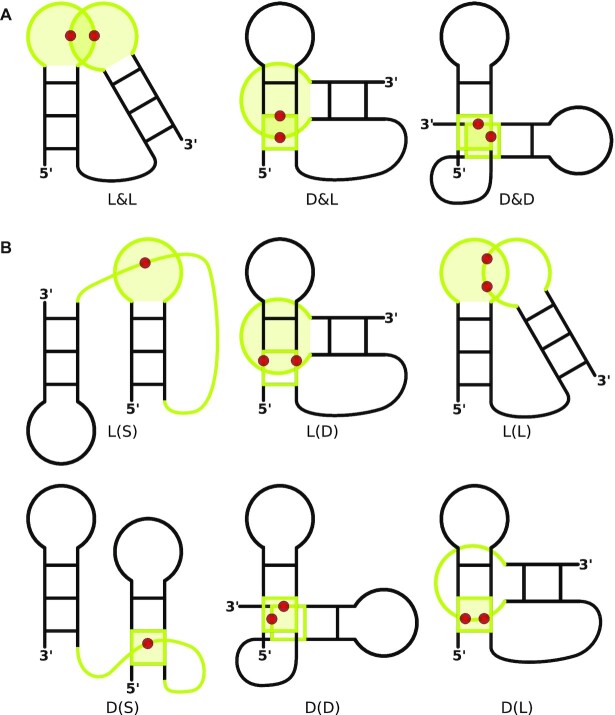
Classification of entanglements of structure elements shown on the example of entangled RNA hairpins: (**A**) interlaces and (**B**) lassos.

**Figure 2.**
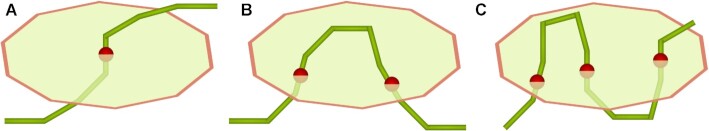
Single strand S punctures a closed element L (**A**) once, (**B**) twice or (**C**) three times, forming L(S), L(S.) or L(S..)-type entanglement.

We distinguish two entanglement topologies (Figure 1). Lasso – in RNAspider encoded using ( ) - consists of elements, in which one is only punctured, while the other only punctures. In interlace, represented by &, they do both to each other. Entangled elements are marked in the system using capital letters—L stands for a loop, S for a single strand, and D for a dinucleotide step. Combining symbols from both groups allows us to describe entanglement classes, e.g. L&D denotes a loop interlaced with a dinucleotide step, L(S) is a loop lassoed over a single strand. The system additionally supports multiple punctures (Figure 2) – dots in the classification scheme represent extra punctures, e.g. L(S..) is a loop lassoed over a single strand that punctures it thrice (Figure 2C).

RNAspider uses the concept of pseudoknot order (32,34) to distinguish between the core structure and the pseudoknotted base pairs. Initially, it only considers the core structure (non-pseudoknotted) and separates it into structural elements. At this stage, RNAspider treats nucleotides involved in pseudoknots as unpaired (so they get embedded inside loops and single strands) and detects *primary entanglements*. Thus, this type of entanglement is formed by structural elements that are not closed by pseudoknotted pairs. Next, the computational engine analyzes the input RNA structure separately for each pseudoknot order (increasingly to the user-selected value). Structural elements are constructed anew based on pseudoknotted base pairs of the current order. This time, nucleotides of the core structure might end up embedded inside. Such iterative procedure allows identifying *higher-order entanglements* defined upon long-distance, pseudoknotted base pairs. It runs by default unless switched off by the user selecting the *Ignoring pseudoknots* option in the advanced settings panel.

## WEB APPLICATION

RNAspider has a multi-layer architecture. The business logic layer integrates a computational module, message broker, and database, providing RESTful API employed by a user-friendly web interface. The computational module is developed using Java 11 with Apache Maven 3.5 and other third-party libraries. It provides efficient data processing, even for large, multi-model RNAs. RabbitMQ, an open-source message broker, ensures that no submitted task is lost. Python 3.6 and Gunicorn support the Flask web application. The database uses PostgreSQL, an open-source, object-relational database management system. Celery, a reliable distributed queuing system, performs cyclic operations like removing expired tasks. The RNAspider UI implemented in React follows the single-page application pattern. It supports all modern browsers and platforms, including mobile devices. Visualization of the RNA structure with entanglements is possible with suitably modified VARNA (35) and Mol* (36) programs. Docker-based containerization minimizes the likelihood of system failure and increases its scalability. RNAspider works on a virtual machine with 16GB RAM and 6 CPU units Intel(R) Xeon(R) Gold 6138 CPU @ 2.00 GHz. It is hosted and maintained by the Institute of Computing Science, Poznan University of Technology, Poland.

### Input and output description

After accessing the RNAspider homepage (Supplementary Figure S1A), users upload input files in PDB or PDBx/mmCIF format. The web application can also load one of the predefined examples or fetch structural data straight from the Protein Data Bank (37) given one or more PDB identifiers. RNAspider accepts input data of the total size not exceeding 50MB per session. For multi-model files, users can set the processing of either the first or all input models. The advanced settings panel allows further tweaking. Here, one can choose to ignore isolated base pairs, ignore or accept pseudoknots when identifying closed structural elements, set maximum loop length (crucial for loops including pseudoknotted base pairs), and specify atoms used to construct the polygonal chain. Finally, users can select to receive email notifications with processing results.

The result page (Supplementary Figure S1B) has a dedicated, bookmarkable URL that allows users to return up to 14 days after completing the task. On its left side, one can find a list of processed files with a note on identified entanglements (*File List* tab). It is possible to download an archive with selected output data: cleaned 3D structure, secondary structure in extended dot-bracket notation, secondary structure diagram, or the result table (*Download* tab). The right panel presents the main findings divided into sections. The top one includes general information and a resubmission form to run data analysis with different parameter values. Below, users can see the RNA sequence and secondary structure in extended dot-bracket notation (31). The following section contains a table of entanglements described by identifier, class, topology, involved structural elements, and puncture sites. Straight from the table, users can download 3D coordinates of entangled elements. Bottom sections present 2D and 3D structure visualizations, color-coded and interactive, allowing the study of every entanglement’s context independently. In the 3D model, a semi-transparent mesh displays closed elements’ surfaces, and red beads depict puncture sites.

## RESULTS AND DISCUSSION

### Examples of entangled structures

Here, we demonstrate the use of RNAspider for two structures obtained for fragments of viral RNA genomes. In the first example, the program allowed the verification of computationally generated RNA 3D models. For the other, it supported the analysis of a complex pseudoknotted structure.

**Figure 3.**
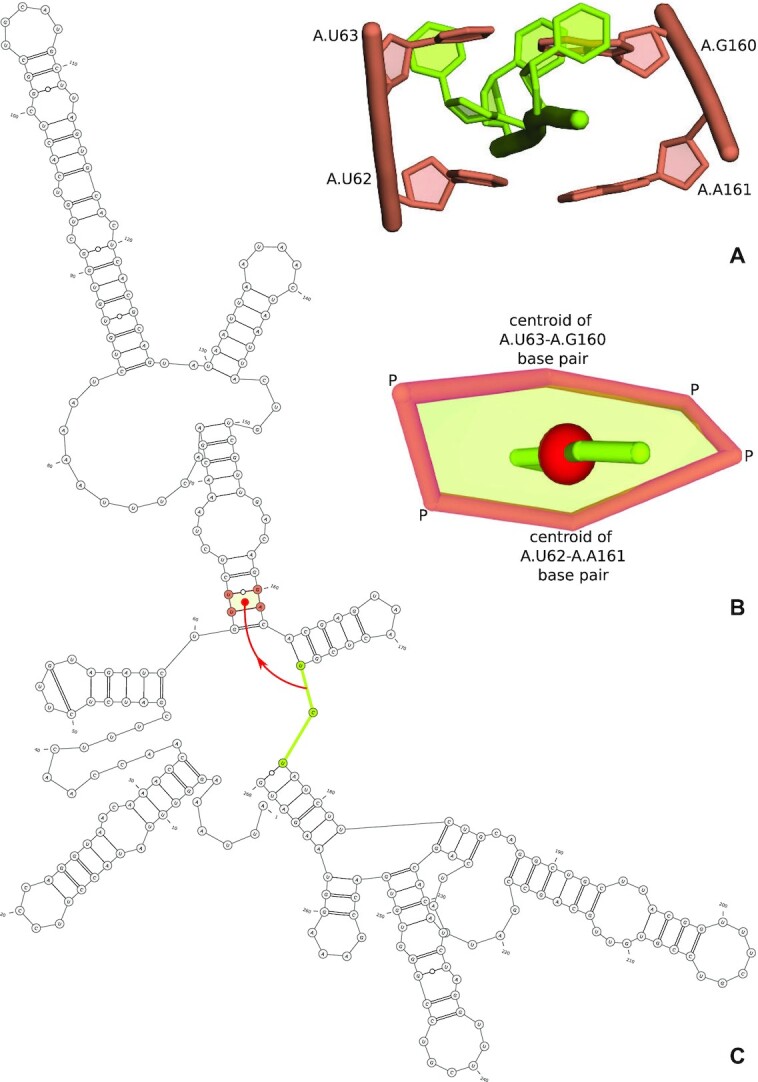
Entanglement of type D(S) –dinucleotide step lassoing the single-stranded fragment – from RNAComposer-5UTR model shown as (**A**) a full-atom representation, (**B**) a simplified view from RNAspider and (**C**) a 2D structure with highlighted elements forming the entanglement.

First, we focused on untranslated regions of the SARS-CoV-2 RNA genome. Structure motifs present in SARS-CoV-2 are crucial for developing RNA-targeted small-molecule drugs. Thus, many researchers have recently attempted to characterize the tertiary structures of selected fragments of this viral genome, both with experimental methods and computational modeling (38,39). Also, the RNA-Puzzles community has challenged the prediction of the 3D structures of functionally essential RNA elements in the SARS-CoV-2 genome, namely the 3’- and 5’-UTR (40). This initiative resulted in an ensemble of 100 RNA models, 25 of which contained entanglements (Supplementary Table S1). The latter include the RNAComposer-5UTR model obtained for the 268 nucleotide-long fragment of 5′-UTR of the coronavirus genome. In this structure, RNAspider identified lasso type D(S) entanglement – the inter-domain single-stranded fragment (U175–U177) punctures the center of the dinucleotide step (U62–U63, G160–A161) (Figure 3). Such entanglement can be treated as incorrect geometry like all interlaces and lassos of type D(S) or L(S) with strand S that connects two helical regions and punctures a dinucleotide step or a loop between these regions. We suggest eliminating such RNA 3D models as unreliable.

**Figure 4.**
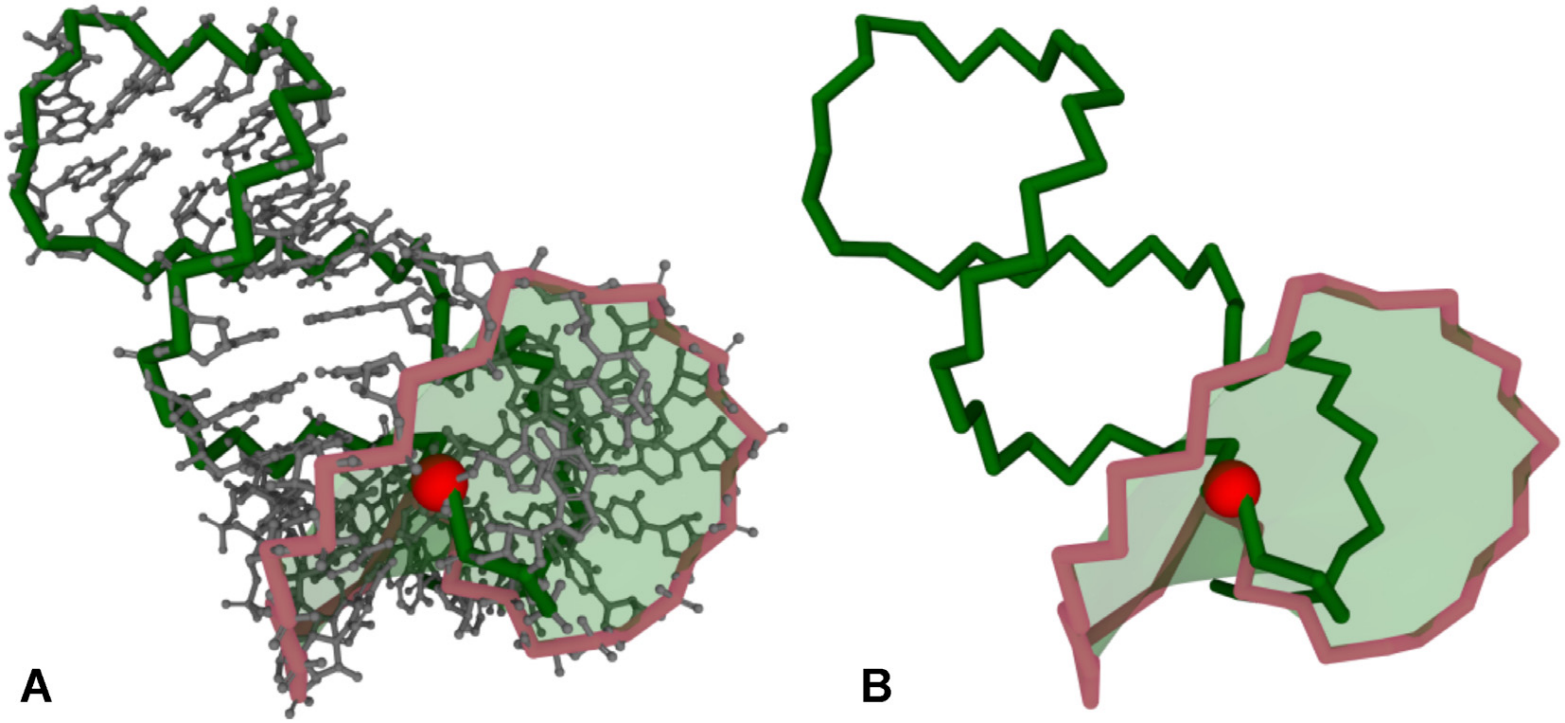
Higher-order L(S) entanglement in a knot-like fold (PDB ID: 7K16) visualized by RNAspider in (**A**) full-atom and (**B**) simplified representation. The red bead indicates the point of puncturing the surface spanned on the loop by the polygonal chain of the 5′-end. The point of intersection falls between the C4′ atom of residue 2 and the P atom of residue 3 of the polygonal chain.

As a second example, we present exoribonuclease-resistant RNA (xrRNA) from the flavivirus molecule. Its structure, determined by X-ray crystallography (PDB ID: 7K16, resolution 2.10 Å) (41), contains an unusual fold, which – in the literature – is referred to as a knot-like fold or ring-like architecture (23,42). Such folds exhibit distinctive mechanical properties as revealed by experimental studies using force spectroscopy (23,24). So far, they have been identified in experimental structures of xrRNAs (PDB IDs: 5TPY, 7K16) and SARS-CoV-2 FSE (PDB IDs: 6XRZ, 7O7Z) (42) as well as in computational models of SARS-CoV-2 FSE (43–46). The formation of a knot-like fold in the structure requires the presence of a pseudoknot. According to the RNAspider classification, the knot-like fold is a higher-order entanglement of the L(S) type – loop lassoing the single-stranded fragment is closed by pseudoknotted base pair(s). Thus, it can be identified if RNAspider runs with *accepting pseudoknots* option turned on (default settings). In the xrRNA structure, the loop – closed by base pair (G33-C49) of the first order – is punctured by the single strand (g1-G31) from the 5′-end, intersection point between C4’ in G2 and P in C3 (Figure 4). Note that 5′-end is classified as a single-stranded element regarding the pseudoknot of the 1st order. Thus, in general, its residues may be involved in non-pseudoknot base pairs or pseudoknots of a different order.

### Entanglements in the PDB

In (25), we discussed two entangled RNA 3D models determined experimentally. Here, to complete the picture of entanglements in experimental structures, we have examined all RNAs available in the Protein Data Bank as of 2021-12-13.

From the initial set of 5804 RNA structures, we have excluded 11 false positives, that is, molecules with overlapping atoms misrecognized as punctures. Among 5793 instances left, we found 1307 (22%) entangled cases. Fifty-three structures have only primary entanglements, 1232 – only higher-order entanglements, and 22 have both types. Analysis of the results indicates a strong correlation between pseudoknot order and the number of entangled structural elements (0.711 Pearson’s and 0.778 Spearman’s correlation coefficient). Also, the size of a molecule affects the formation of entanglements – the Person's correlation coefficient equals 0.812.

A total of 120 primary entanglements were recognized in 72 RNAs (Table 1). 88% are lassos, usually of the L(S) type. Only 12% constitute interlaces, mainly from the L&L class. 3308 higher-order entanglements were found in 1254 structures from the input set. They consist almost entirely of the L(S) type arrangements. Detailed results of this analysis are available in Supplementary Table S2.

**Table 1.** The number of entanglements identified by RNAspider in the analyzed set of PDB structures

Class	Lassos:	L(S)	L(D)	L(L)	D(S)	D(D)	D(L)	Interlaces:	L&L	D&L	D&D	All
Primary entanglements	106	47	16	31	11	0	1	14	10	3	1	120
Higher-order entanglements	3308	3306	2	0	0	0	0	0	0	0	0	3308
Total number	3414	3353	18	31	11	0	1	14	10	3	1	3428

We hypothesize that interlaces and some lassos are incorrect geometries in molecule 3D models. However, PDB structures containing them should not be unreflectively considered wrong. We suggest their careful analysis to draw correct conclusions. For example, some entanglements in experimental structures may result from two molecules having alternative conformations.

## CONCLUSIONS

RNAspider is the first bioinformatics tool that automatically identifies, classifies, and visualizes entanglements in RNA 3D structures. Such uncommon arrangements of structural elements – taking on various topologies – arise when one of the elements punctures the area defined by the other. In (25), we showed that they appear in computer-predicted RNA 3D models. Here, we examined all PDB-deposited RNA structures to find that 1% contain primary entanglements, which can be used as a rule of thumb in an evaluation of RNA 3D models. 22% of the analyzed structures have higher-order entanglements between elements defined by long-distance, pseudoknotted base pairs. They include recently examined knot-like fold prevalent in xrRNAs and SARS-CoV-2 FSE.

## Supplementary Material

gkac218_Supplemental_Files
