# Supplementary material for: RNAspider: a webserver to analyze entanglements in RNA 3D structures
Source: Nucleic Acids Res. 2022 Mar 29;50(W1):W663–9. doi: 10.1093/nar/gkac218 (PMC9252836; doi:10.1093/nar/gkac218)
Supplement: gkac218_Supplemental_Files [file gkac218_supplemental_files.zip › RNAspider-Supplementary-Material.pdf]

Supplementary Material

RNAspider: a webserver to analyze entanglements in RNA 3D structures

Kamil Luwanski[1], Vladyslav Hlushchenko[1], Mariusz Popenda[2], Tomasz Zok[1],

Joanna Sarzynska[2], Daniil Martsich[1], Marta Szachniuk[1,2,*], Maciej Antczak[1,2,*]

[1] Institute of Computing Science and European Centre for Bioinformatics and Genomics, Poznan University of Technology, 60-965 Poznan, Poland

[2] Institute of Bioorganic Chemistry, Polish Academy of Sciences, 61-704 Poznan, Poland

*To whom correspondence should be addressed:

mszachniuk@cs.put.poznan.pl, mantczak@cs.put.poznan.pl

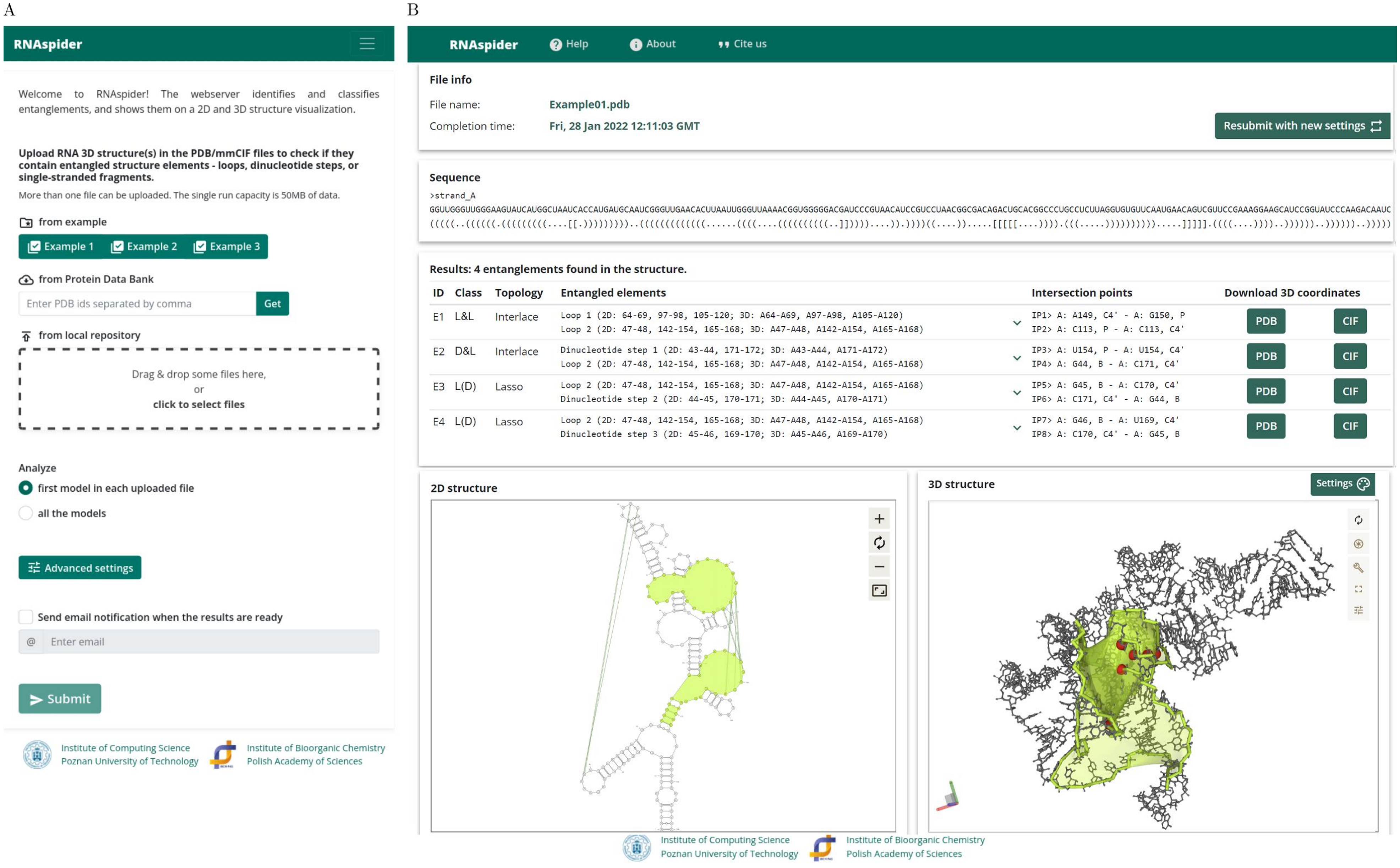

Figure S1. RNAspider user interface. (A) Input form on the home page. (B) Output page.

Table S1. RNAspider results for predicted models of 5'- and 3'-UTR of SARS-CoV-2 structure.

Region of SARS-CoV-2 genome	Sequence length	Number of RNA 3D models	Number of entangled models	Models with entanglements	Simple entanglements	Higher-order entanglements
5' UTR	268	26	4	RNAComposer-5UTR	D(S)	
				5UTR-Miao-02	L(S)	
				5UTR-Miao-03	D&L	
				5UTR-Miao-04	L(S), L(S.)	L(S)
5' UTR extended	293	34	2	5UTR-Bujnicki-02		L(D), L(S)
				5UTR-Bujnicki-05		L(D)
3' UTR with pseudoknot	328	15	15	3UTR-Chen-2_1		L(S)
				3UTR-Chen-2_2	L(S)	L(S)
				3UTR-Chen-2_3	L(S)	L(S)
				3UTR-Chen-2_4	L(S)	L(S)
				3UTR-Chen-2_5	L(S)	L(S)
				3UTR-Das-01_1	L(S)	L(S)
				3UTR-Das-02_1	L(S)	L(S)
				3UTR-Das-03_1		L(D), L(D), L(S)
				3UTR-Das-04_1	L(S)	L(S)
				3UTR-Das-05_1	L(S)	L(S)
				3UTR-Das-06_1	L(S)	L(S)
				3UTR-Das-07_1	L(S)	L(S)
				3UTR-Das-08_1	L(S)	L(S)
				3UTR-Das-09_1	L(S)	L(S)
				3UTR-Das-10_1	L(S)	L(S)
3' UTR without pseudoknot	337	25	4	3UTR-Bujnicki-03	L(L), L(D)	
				3UTR-Bujnicki-04	L(L), L(D)	
				3UTR-Bujnicki-05	L(L), L(D), L(D)	
				3UTR-Ding_7	L(L), L(D)	
